# Chemical and Microstructural Characterization of pH and [Ca^2+^] Dependent Sol-Gel Transitions in Mucin Biopolymer

**DOI:** 10.1038/s41598-020-65392-4

**Published:** 2020-05-29

**Authors:** Austin Curnutt, Kaylee Smith, Emily Darrow, Keisha B. Walters

**Affiliations:** School of Chemical, Biological and Materials Engineering, University of Oklahoma, Norman, Oklahoma 73019 USA

**Keywords:** Biophysics, Single-molecule biophysics, Materials science, Biomaterials - proteins

## Abstract

Mucus is responsible for controlling transport and barrier function in biological systems, and its properties can be significantly affected by compositional and environmental changes. In this study, the impacts of pH and CaCl_2_ were examined on the solution-to-gel transition of mucin, the primary structural component of mucus. Microscale structural changes were correlated with macroscale viscoelastic behavior as a function of pH and calcium addition using rheology, dynamic light scattering, zeta potential, surface tension, and FTIR spectroscopic characterization. Mucin solutions transitioned from solution to gel behavior between pH 4–5 and correspondingly displayed a more than ten-fold increase in viscoelastic moduli. Addition of CaCl_2_ increased the sol-gel transition pH value to ca. 6, with a twofold increase in loss moduli at low frequencies and ten-fold increase in storage modulus. Changing the ionic conditions—specifically [H^+^] and [Ca^2+^] —modulated the sol-gel transition pH, isoelectric point, and viscoelastic properties due to reversible conformational changes with mucin forming a network structure via  non-covalent cross-links between mucin chains.

## Introduction

Mucin is a polyelectrolyte glycoprotein found in mucus, the structured complex fluid found in all types of organisms from bacteria to humans. In vertebrates, mucus is produced by mucus membranes and can be found lining the eyelids, mouth, and nose, as well as gastrointestinal, respiratory, and genital tracts. Since the 1970s^1^, animal mucus and mucin solutions have been well studied, especially the roles mucus plays in drug delivery^2–6^, disorders like cystic fibrosis^7,8^, and its protective biological functions^9–12^. Mucin glycoproteins are present in mucus at concentrations of 1–5%, along with electrolytes (ca. 1%), lipids (1–2%), other proteins (1–2%), and water (90–95%)^13^. Despite being present in low concentrations, mucin glycoproteins are primarily responsible for the protective and lubricative functions of mucus within the body.

Solubilized mucin behaves as a complex fluid with changing viscoelastic and structural properties in response to its environmental conditions. Mucin glycoproteins are responsible for the majority of the physical properties of mucus, as mucin conformation, intra- and inter-strand bonding, and microstructure changes in response to environmental factors such as pH^6,7,12,14^, temperature^2,15^, and ion content^1,14,16^. All these factors affect the reversible supramolecular bonding between functional groups within the protein strands, which modifies the microstructure as well as the mechanical and transport properties of the mucin network. In aqueous solutions, biopolymers like mucin form networks that can change dynamically due to non-covalent crosslinks, including physical entanglements and supramolecular bonding. Mucins resemble a bottle brush polymer with a protein backbone and oligosaccharide (carbohydrate) side chains arranged radially from the backbone. The protein backbone of the mucin biopolymer has areas that are dense with cysteine, carboxyl, and amine functional groups and other regions that are dense with proline, threonine, and serine where there is O-linked glycosylation. The glycosylation of mucin can be so extensive that 80% of the mucin mass is comprised of the oligosaccharide sidechains and the remaining 20 wt% is the protein core^12^. Therefore, the electrostatic state of the side chains plays a dominant role in mucin biopolymer intra- and inter-strand interactions, conformation, and the ultimate ability of mucin to form a network structure.

**Figure 1:**
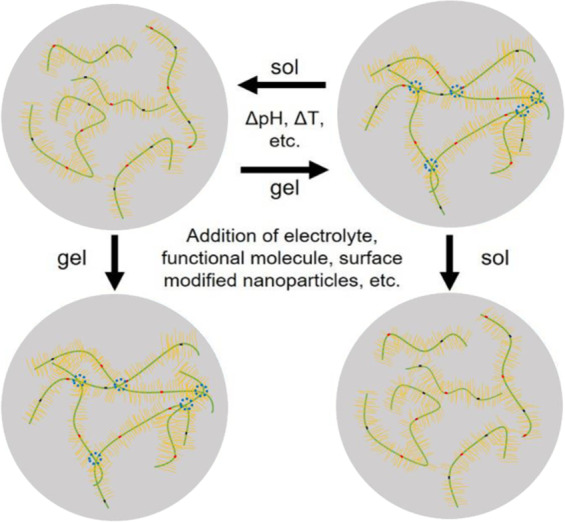
Sol-gel transitions can take place in structured fluids in response to changes in environmental factors such as pH, ion concentration, inclusion/particle chemistry and size, temperature, and shear forces. Gels form when the self-affinity of solubilized material increases to form a network structure while remaining soluble. Green lines represent mucin protein cores and yellow lines represent oligosaccharide side chains. In addition to disulfide bonding at the ends of individual mucins (red and black dots), physical and chemical non-covalent cross-linking occurs between mucins in the gelled state (blue circles).

Gel hydration also plays an important role in mucus properties^17,18^, as does the size(s)^19,20^ and surface chemistry^4,9,10,21–23^ of any non-native materials present. The ability for mucin solutions to reversibly form a gel is important for controlling the barrier properties of mucus and impacts disease protection, lubrication, and drug delivery efficacy. The transition from solution state to gel state is accompanied by changes in interactions between mucin strands, physical structures present in solution, and mechanical properties. As shown in Fig. 1, the sol-gel transition in mucin solutions is reversible, and the transition point can be modulated using parameters such as pH, temperature, and ionic strength.

Native mucus, especially non-reconstituted, can be difficult to obtain without regular access to human or animal subjects, so porcine gastric mucin (PGM) solutions are often used as a model for mucus studies. PGM is relatively easy to extract and widely available commercially. In addition, PGM solutions are generally considered a viable system to examine mucus sol-gel behavior due to similarities in physiological function^12,24,25^, although differences between native mucus and PGM solutions were observed in a prior study examining nanoparticle transport^26^. In addition to availability and physiological similarity, another critical benefit in using PGM solutions for fundamental studies  is removal of variations in mucin concentration, type, and extraction method, as well as patient to patient variation that occurs with native mucus sources^27,28^. By eliminating variability in the mucin composition, the physicochemical properties of mucin, including interactions between mucin, effects of pH and salt on the sol-gel transition, rheology, and multi-scale structures, can be more effectively studied.

An examination of the literature provides several studies that each examine isolated aspects of mucus and mucin properties, but these properties are often not connected, especially within the same body of work. The microstructure and mechanical properties of mucin have been characterized using rheology^6,29,30^, light scattering techniques^15,31^, zeta potential^32,33^, infrared spectroscopy^32,34^, and various other techniques. Of these methods, IR spectroscopy is the most recently used tool (as it was not used prior to 1995^24^) and shows promise for illustrating supramolecular interactions between glycosylated domains in mucin^35^. The new FTIR results presented here regarding mucin glycosylation provide support for historical and the more recent findings of Meldrum *et al*. regarding the role of Ca^2+^ in mucin gelling behavior^1,30,36,37^.

In the past 20 years, many studies have sought to understand the complex relationships between the chemical and structural properties of mucin by focusing on a single characterization technique. This study is intended as an effort to characterize and correlate micro- and macro-scale properties of mucin during the sol-gel transition by examining the results of pH and ion effects over multiple length scales. Mucin viscoelastic behavior was examined using oscillatory frequency sweep and flow sweep rheology experiments, and the storage and loss moduli and viscosity were measured as a function of pH and the addition of CaCl_2_. In addition to rheological characterization, structural properties related to mucin-mucin interaction and the sol-gel transition were studied using zeta potential, dynamic light scattering, surface tension, and Fourier transform infrared spectroscopy. Average micro-domain size and size distribution data were coupled with changes in net surface charge, interfacial tension, and functional group interactions to examine the how the microstructural changes observed with rheology could be correlated to molecular-level changes. Structure-property changes related to the sol-gel transition for PGM were examined with regard to ion effects and pH value. A better understanding of the relationship between the viscoelastic behavior of mucin and its dynamic network structure is critical for predicting and controlling mucus properties, which can aid in designing therapeutic treatments for diseases involving mucus.

## Results and Discussion

### Mucin rheological behavior and microstructure as a function of pH and [Ca^2+^]

Electrostatic and hydrophobic interactions play a critical role in the conformation, solvency, substrate interactions, and barrier properties of proteins^13,24,38^. Modulating pH, ionic strength, and ion valencies allows for the ionic state of the solution to be adjusted^3,12,13^, thereby allowing for control over sol-gel transitions in mucus and in mucin solutions^1,13,39^. Studies have shown that multivalent cations can affect mucin conformations even at very low concentrations (~1 mM)^1,36,39^. Unlike the electrostatic screening observed with monovalent ions, there is evidence that multivalent ions bind with specific anionic sites on the mucin side chains. For example, in cystic fibrosis increased calcium (Ca^2+^) concentration is known to increase mucus density and viscosity and decrease mucin solubility^7,36^. Therefore, the addition of calcium chloride was studied to examine the effect of divalent cations on mucin solution rheology and microstructure.

#### Flow sweep rheology characterization

**Figure 2:**
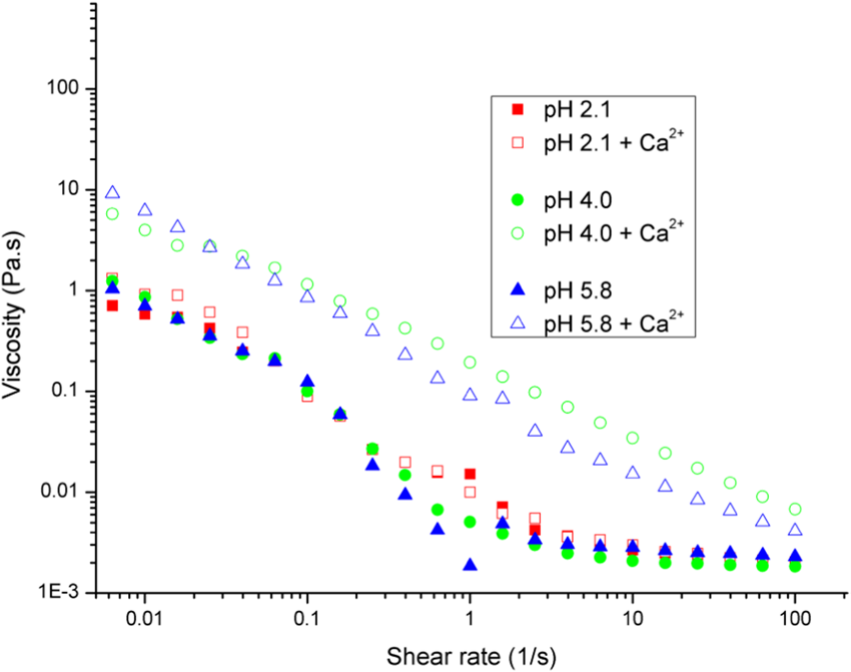
Flow sweeps over a shear rate of 0.001–100 s^−1^ show samples at pH 2.1, 4.0, and 5.8 with 10 mM CaCl_2_ display shear-thinning behavior with a more linear response than samples without CaCl_2_.

Rheology flow sweep measurements were collected on 10 mg/mL PGM solutions at 25 °C over shear rates from 0.01 to 100 s^−1^ (Fig. 2). PGM solutions were examined at three discrete pH values (2.1, 4.0, and 5.8) both without and with 10 mM CaCl_2_ (denoted as PGM + Ca^2+^). Results show all PGM solutions exhibit non-Newtonian, shear-thinning behavior with lower viscosities at higher shear rates. At shear rates greater than ca. 1 s^−1^, the slope changes and the solution viscosities approach 0.002 Pa-s, indicating the minimum viscosity was approached under progressive network deformation^40,41^. The shear thinning behavior of mucin solutions was expected based on prior work by the authors and is supported by previous work by other researchers^6,27,28,42^. Physiologically this rheological feature allows for better clearance of mucus during high shear processes like inhalation and coughing^43,44^. The trends in viscosity behavior as a function of pH, the slope of viscosity vs. shear rate data, and the change in slope of the viscosity data at higher shear rates all align with the study by Celli *et al*.^6^ using 15 mg/mL solutions of commercial mucin. Also examined in the present study are the combined effects of changing the H^+^ ion concentration in concert with the presence of Ca^2+^ ions. As shown in Fig. 2, the addition of a small concentration of calcium results in a significant (ca. 10-fold) increase in viscosity for the pH 4 and 5.8 mucin solutions. For these solutions, the concentration of H^+^ ions is low enough that the addition of Ca^2+^ causes more intermolecular associative interactions resulting in a higher dynamic viscosity and less pronounced slope^45^. For the pH 2.1 mucin solution, only a slight viscosity increase is observed with Ca^2+^ addition at lower shear rates. Due to the large concentration of H^+^ ions present at low pH, the addition of Ca^2+^ does not have a significant impact on shear viscosity due to charge screening.

Prior studies have shown that non-covalent mucin–mucin interactions are pH sensitive and regulated by charge interactions. The H^+^ concentration determines both the net charge of glycosylated domains and the hydration state of mucins^46^. Both high H^+^ concentration and the presence of other ions, including Ca^2+^, impact intramolecular bonding, but do so independently^7^. The effect of Ca^2+^ on mucin rheology provides insight into the role of calcium in mucin strand organization and structure. This finding is significant as calcium is known to play a strong role in mucus secretion, as well as intergranular mucin storage^47^, which is dependent on mucin inter- and intra-strand associations involved in folding (conformation changes) and the network formed in the gel state. Calcium ions affect the expansion and condensation of polymeric mucins by shielding the negatively charged sulfate and sialic acid groups^48–51^. While prior work on the swelling of granular mucin matrices showed decreased velocity of swelling for low pH combined with the presence of calcium^16^, this relationship had not been demonstrated in mucin solutions and the impact on sol-gel transition and viscoelastic behavior had not been demonstrated.

#### Oscillatory sweep rheology characterization

Understanding how molecular-level functional group interactions impact the macroscale rheological behavior of mucin solutions provides insight into polyelectrolyte solution physics and multiscale hierarchical and structured fluids. This insight may even be applied to designing pulmonary therapeutics and drug delivery mechanisms where modulations in physiological ionic conditions are important. Oscillatory rheology experiments allow for the examination of the storage and loss moduli separately due to their different time constants. The storage modulus (G′) relates stress to strain and gives a measure of the resistance to stretching, while the loss modulus (G′′) relates the strain to the time after stress is removed and gives a measure of viscous flow. In addition, for the weak sol-gel network system in mucins, oscillatory rheology can help show structure changes as long as the strain remains low enough to not disrupt the chemical or physical processes responsible for the structure.

**Figure 3:**
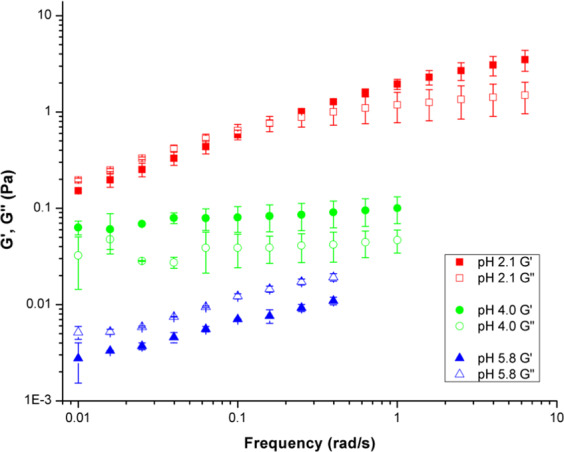
Aqueous PGM solutions display pH-dependent sol-gel behavior and moduli, G′ (closed symbols) and G′′ (open symbols). Oscillating frequency sweep rheology data are shown over 0.01–10 rad/s for pH values of 2.1, 4.0, and 5.8.

Oscillatory sweeps were performed over a low frequency range to further characterize PGM solution sol-gel transition and viscoelastic behavior. Three frequency sweeps were performed on PGM solutions at pH 2.1, 4.0, and 5.8 and resulting averages are shown in Fig. 3. Even at low shear rates, the trends observed do not match the flow sweep measurements. While complex viscosity data can be calculated from the moduli data of frequency sweeps, the viscosity data from the flow sweeps (Fig. 2) cannot be expected to have the same trends as the complex viscosity data from the frequency sweeps. The two sets of data are not comparable because the shear strain rates used in each type of test are not constant. The applied strain amplitude in the oscillatory experiments may not be low enough to remain in the linear viscoelastic region (LVR). For mucin solutions, the dynamic viscosity is greater than the steady shear viscosity, indicating that the Cox-Merz rule is not applicable^40^. This behavior is common for systems that gel or contain particulate dispersions due to dispersed phase interactions and/or network deformation^40,41^.

In Fig. 3, there is a clear change in the viscoelastic moduli in response to changes in pH. The pH 2.1 solution displays a well-structured gel-like behavior with G′ and G′′ crossover occurring mid-way in the frequency sweep and G′ > G′′ maintained from 0.1 to 6 rad/s. This mid-frequency transition was similarly observed for increased solids concentration (instead of increased H^+^ concentration as in our study) along with the leveling off of the G′′ slope at higher frequencies^40^. The similar rheological behaviors for higher mucin and H^+^ concentrations is likely due to the increase in polymer-polymer interactions dominating the viscous forces^30,40^. The overlap of G′ and G′′ for the pH 2.1 sample at frequencies below 1 rad/s may be due to instrument resolution as the strain amplitude was ca. 0.003, 0.02, and 0.2 for pH 2.1, 4.0, and 5.8 respectively. However, this G′ and G′′ overlap at low frequencies was also observed for mucin at 1 wt% and pH 2 by other researchers, and, like our findings, they also observed that, at pH values above the critical sol-gel transition pH, pH_G_, both G′ and G′′ decreased^52^. The pH 4 solution also shows gel-behavior with G′ > G′′. The moduli insensitivity to frequency at pH 4 is indicative that the sample is at or near the pH_G_ as this rheological characteristic is indicative of a shear gel containing micro-domains^30^. As pH increases further past the pH_G_, the concentration of H^+^ ions decreases to the point the gel structure is lost and the system exhibits solution-like behavior with G′′ > G′. Solution behavior (G′′ > G′) is clearly observed in the pH 5.8 sample. Our findings indicating a pH-sensitive sol-gel transition in PGM aqueous solutions align well with previous work^6,30^, and mechanisms related to ionic polymer-polymer interactions can be extended to concentration induced polymer-polymer interactions in the non-dilute regime^30,40^. The change in the storage (elastic) and loss (viscous) moduli as a function of pH in these dilute solutions indicate the H^+^ concentration impacts the macro-scale network structure through changes in micro-domain structures^12,30,40,53^. The network structure is able to dynamically form via non-covalent supramolecular mucin-mucin interactions which will be probed further using chemical and physical characterization methods with and without Ca^2+^ addition.

**Figure 4:**
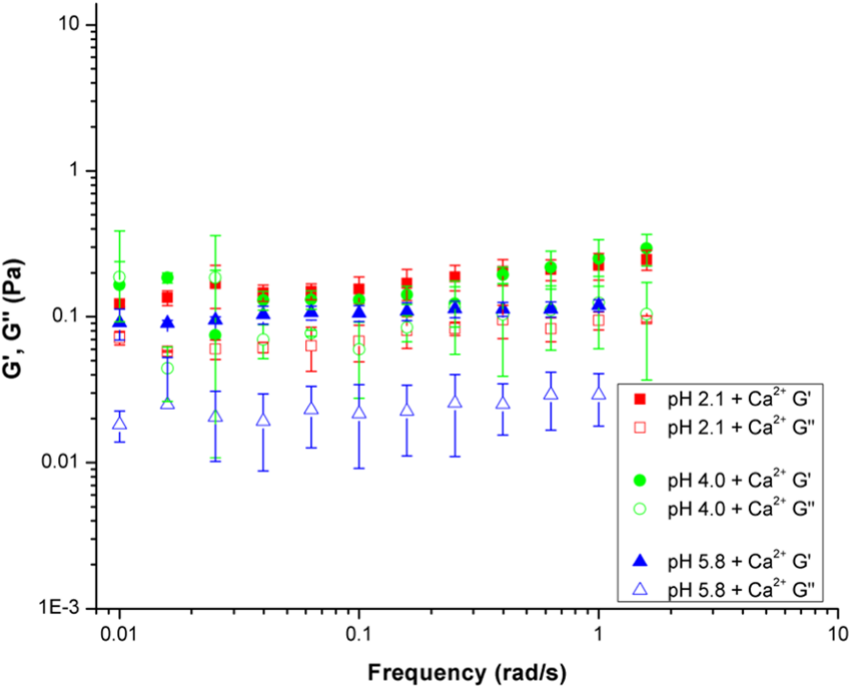
Oscillating frequency sweep rheology data over 0.01–2 rad/s for aqueous PGM solutions at pH values of 2.1 (square), 4.0 (circle), and 5.8 (triangle), with 0.01 M calcium chloride added.

Oscillating frequency sweep rheological experiments were also performed on PGM solutions at pH 2.1, 4.0, and 5.8 with 10 mM CaCl_2_ added (Fig. 4). As discussed previously, the pH-dependent sol-gel transition, pH_G_, takes place at ca. pH 4 for PGM solutions. The frequency sweep data in Fig. 4 shows the effect of Ca^2+^ on the pH_G_ of PGM. Upon the addition of calcium chloride, the pH 2.1 PGM solution moduli decreased indicating disruption of the mucin network structure—with a significant decrease in the loss modulus (G′′) which is a measure of viscous response. PGM samples with CaCl_2_ added have G′ > G′′ for all pH values tested above 0.1 rad/s (Fig. 4). Therefore, all samples are in a gel-like state due to the presence of Ca^2+^. The error bars in Fig. 4 provide information on sample-to-sample variation, and the large size of these error bars is to be expected for a complex, amphiphilic biopolymer. However, the trend of G′ > G′′ can be clearly observed. The addition of Ca^2+^ also increased the strength of the weak polymer network, as the moduli become insensitive to frequency. At pH 4.0 and 5.8, the PGM solution moduli increased with the addition of CaCl_2_. This increase in modulus points towards the development of a stabilizing mechanism in which the mucins were able to bind more strongly with one another. Although viscosity increases with larger moduli, determination of gel-like behavior is still dependent on the relative values of G′ and G′′. Gel-like behavior occurs when G′ is greater than G′′. While both pH 4.0 and pH 5.8 PGM samples showed an increase in G′ and G′′ with the addition of calcium chloride, only the pH 5.8 sample showed a transition from solution- to gel-like behavior. It is likely that the pH 4.0 sample was at the border of the gel regime and displayed both solution and gel behaviors. The overall behaviors shown in both Figs. 3 and 4 indicate that CaCl_2_ addition suppresses the effect of H^+^ concentration.

#### Examining storage and loss moduli

**Figure 5:**
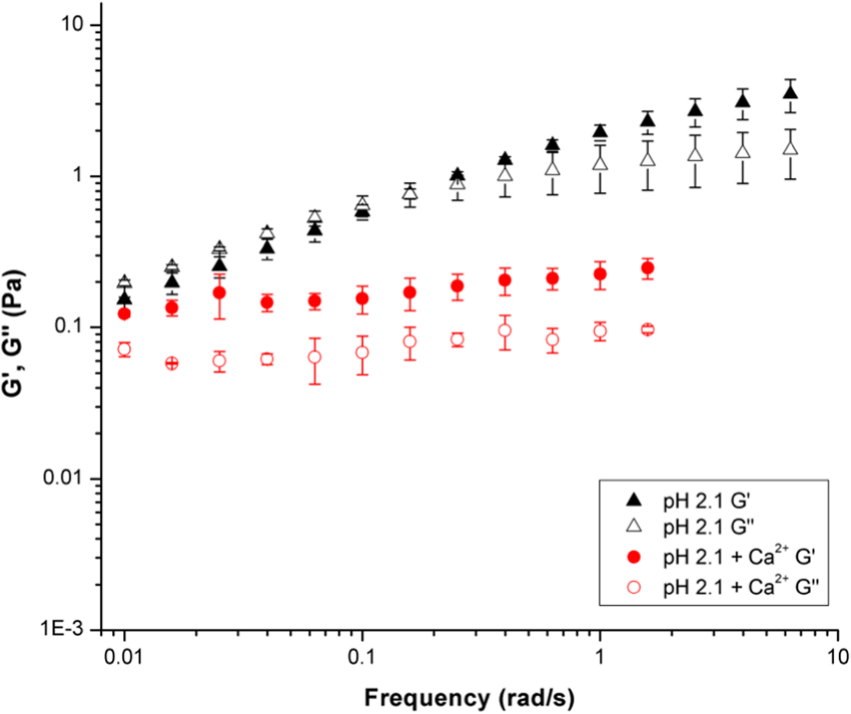
Storage (G′) and loss (G′′) moduli of PGM solutions at pH 2.1 are shown to decrease with the addition of 10 mM calcium chloride.

The PGM mucin solutions at pH 2.1 are in the gelled regime (Fig. 5), as exhibited by the dominant storage moduli in both samples (without Ca^2+^, this is seen above 0.1 rad/s). In this regime, the addition of 10 mM calcium chloride caused a decrease in both the storage and loss moduli. The storage modulus continues to dominate the loss modulus, indicating gel behavior even with CaCl_2_ present. Note that in gels, the difference between G′ and G′′ stays approximately constant as a function of frequency until the network structure is disturbed. Divalent ions, such as Ca^2+^, are known to reduce swelling in mucin gel networks at low pH, as the acidic groups in the carbohydrate side chains become less charged exposing hydrophobic sites. These hydrophobic interactions form non-covalent cross-links between mucins that lead to and maintain the gel state^5,25,31,54^.

**Figure 6:**
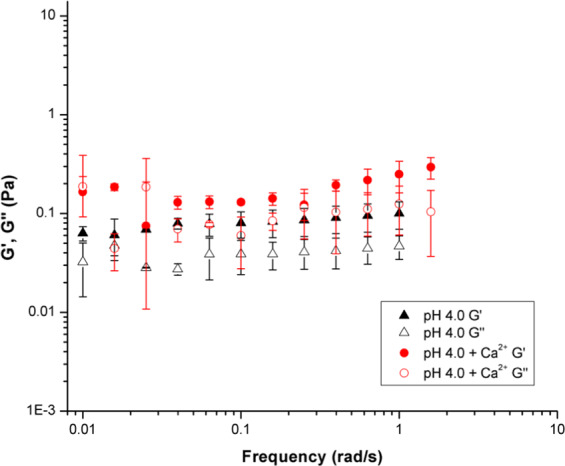
Storage (G′) and loss (G′′) moduli of PGM solutions at pH 4.0 increase with the addition of 10 mM calcium chloride.

**Figure 7:**
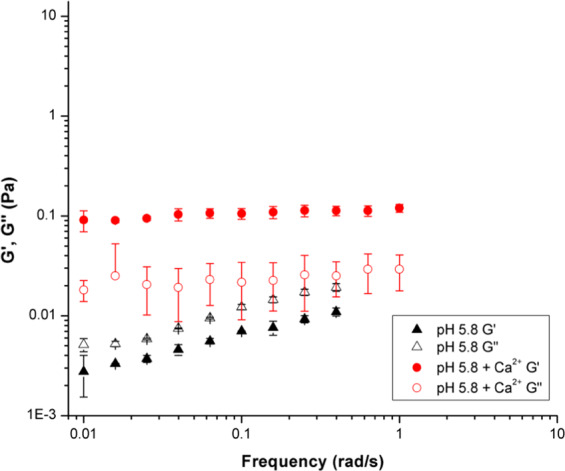
Storage (G′) and loss (G′′) moduli of PGM solutions at pH 5.8 increase with the addition of 10 mM calcium chloride.

The solutions at pH 4.0 (Fig. 6) are also in the gelled regime, as seen by the dominant storage moduli. However, in this case, both the storage and loss moduli of the samples increase with the addition of 10 mM calcium chloride. This behavior suggests that the addition of CaCl_2_ may promote network formation by increased supramolecular interactions between PGM polymer strands—including interactions between glycosylated domains, hydrophobic and electrostatic interactions, and disulfide linkages. Examining Fig. 7, the PGM solution at pH 5.8 shows solution-like behavior in the absence of calcium chloride, as indicated by the loss modulus dominating the storage modulus. With the addition of calcium chloride, the pH 5.8 PGM sample—like the pH 4.0 solution—showed an increase in both moduli. However, unlike the pH 4.0 PGM samples, pH 5.8 PGM exhibited a transition to the gel regime upon addition of CaCl_2_, as shown by the dominant storage modulus.

At higher pH values closer to neutral (ca. 5–7), PGM molecules are largely electronegative. Their non-glycosylated domains are arranged with salt bridges between carboxylates and positively charged amino acids (pKa values near 4) enfolded around the hydrophobic regions of PGM^12^. In the neutral pH regime, the addition of positively charged Ca^2+^ can disrupt the salt bridges, exposing hydrophobic regions which promote the formation of micro-domains within the mucin matrix via non-covalent cross-links (associations). This finding is supported by previous work by Raynal *et al*. which demonstrated a significant increase in the molecular weight and viscosity of salivary mucin in the presence of 10 mM CaCl_2_^37^. At pH values below the pKa (≤ 4), mucin network formation (i.e., gelation) is promoted by non-covalent cross-links via hydrophobic association of newly exposed hydrophobic domains.

#### Examining mucin microstructure with light scattering

In mucin solutions, micro-domains of local order can form from physical or chemical associations between sections of the mucin chains that can lead to polymer network structures^55^. The state of mucin strands in solution and development of these micro-domains and network structure can be analyzed using dynamic light scattering (DLS), which measures average particle (micro-domain) sizes and size distributions. In the present study, the average hydrodynamic particle diameter and size distributions measured using DLS were compared for PGM solutions with and without the addition of CaCl_2_ added and as a function of pH. The impact of mucin concentration on ordered micro-domain formation was not studied due to concentration limitations of the DLS method, which requires samples to be dilute and clear. The 10 mg/mL PGM solutions examined using rheology were opaque and would have resulted in inaccurate particle size measurements if not diluted. Therefore, dilute mucin solution samples (0.1 mg/mL) were examined with DLS. It should be noted that this concentration is below the critical entanglement concentration^30^ and the formation of micro-domains is primarily due to chain-chain supramolecular interactions and not entanglement.

**Figure 8:**
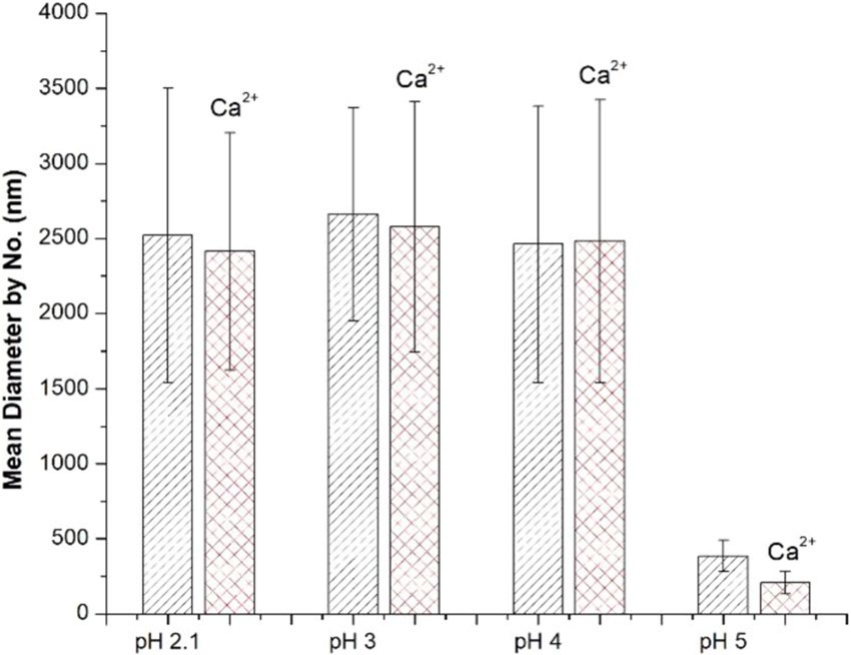
Average PGM hydrodynamic diameters determined by DLS reveal a sharp decrease in effective particle size at pH 4 and above, regardless of CaCl_2_ addition of 10 mM.

DLS data was analyzed to determine the dominant peak, in terms of number of micro-domain particles present. The dominant DLS peak was used as a sample metric for ‘average’ micro-domain particle size. These data display a clear and sharp increase in micro-domain size at pH values ≤4 (Fig. 8). Results from studies by other researchers^6,31,56^ support the results of this study demonstrating that PGM has a pH_G_ value between pH 4 and pH 5. The particle size data in Fig. 8 clearly illustrate the structural changes associated with the sol-gel transitions—as the average micro-domain particle diameter increases approximately five-fold at pH values of 4 and below. This increase in effective micro-domain particle size is the result of increased interactions between adjacent PGM strands. Micrometer-sized micro-domains may be formed upon dilution of the sample, which agrees with previous work by Bromberg and Barr^57^. The mucin-mucin interactions discussed here are due to multiple supramolecular interactions including glycosylation^58^, hydrophobic and electrostatic interactions^31,59^, and disulfide end-to-end linkages^57,60^, which are known to increase in highly acidic environments^61^. The increase in micro-domain size observed with DLS at and below pH 4 indicates the formation of long-range network structure. This finding corroborates the increase in viscoelastic properties observed with rheology in this pH range.

#### Impact of mucin interactions and network formation on surface tension

**Figure 9:**
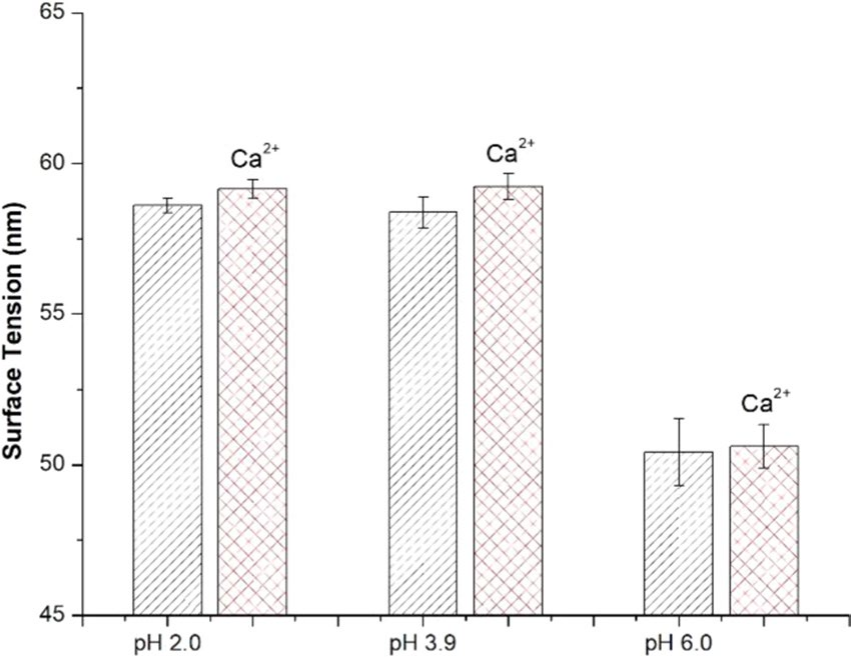
PGM solutions at pH values below the pH_G_ (~ pH 4) exhibit significantly higher surface tension values. Addition of 10 mM CaCl_2_ showed a negligible effect on the surface tension.

Surface tension measurements of PGM solutions at pH 2.0 (n = 4), pH 3.9 (n = 4), and pH 6.0 (n = 3) are presented in Fig. 9. Surface tension data show a clear response to pH with surface tension decreasing over 15% at pH values above the pH_G_. At pH 6.0 the system is clearly in the solution regime. At pH 3.9 and 2.0, the increase in surface tension shows that acidification increases the strength of the gel network^30^ despite observing only minimal increases in the moduli, indicating the mucin network may show preferential organization at the liquid-air interface. These surface tension results support the findings from DLS particle sizing, showing decreased network micro-domain structure above pH 4. At pH values above the pH_G_, the mucin strands associate less with one another through non-covalent hydrophobic cross-linking, resulting in smaller domain sizes, lower moduli, and lower surface tension values.

#### Surface charge effects

**Figure 10:**
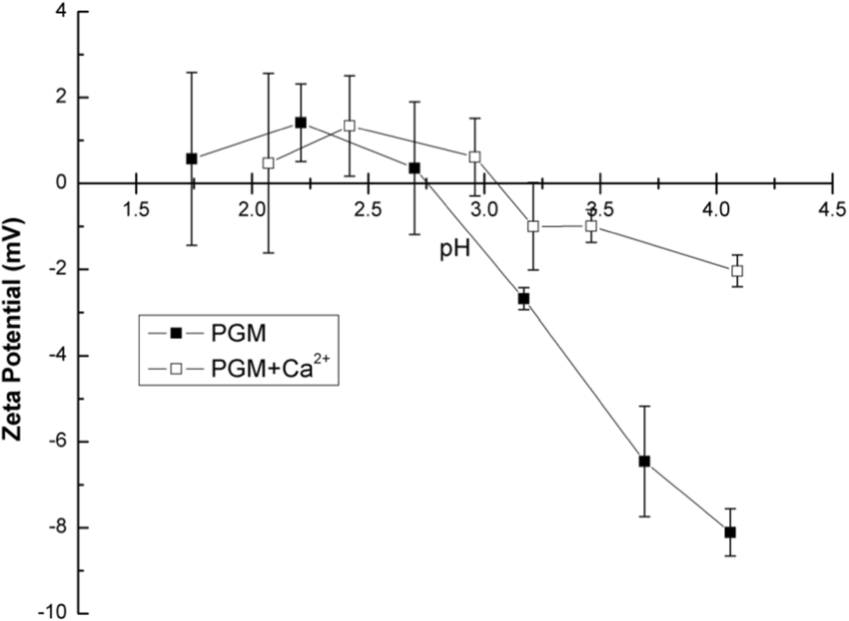
Zeta potential data show PGM solutions have a higher isoelectric point when 10 mM CaCl_2_ is present.

Both electrostatic and hydrophobic interactions of the mucin dictate gelling behavior. Therefore, the effect of pH and CaCl_2_ addition was examined for PGM solutions using zeta potential, a method to measure net surface charge of materials in dilute solutions. PGM micro-domains in aqueous solutions typically have a net negative surface charge at pH values of 3 and above^32,62^. Zeta potential measurements were taken at discrete pH values ≤ 4 to examine the effects of H^+^ and Ca^2+^ concentration. Zeta potential data are plotted as a function of pH in Fig. 10. Comparing the results of the neat PGM solution to PGM + Ca^2+^ reveals that the addition of CaCl_2_ increases the PGM isoelectric point (pI)—where the net surface charge is zero (zeta potential is 0 mV)—from ca. pH 2.75 to pH 3.1. With the addition of CaCl_2_, an increase in pI implies that electrostatic forces are neutralized at a higher pH value with the addition of CaCl_2_. This increase in pI with CaCl_2_ addition supports that the changes in rheological behavior of the PGM solutions were due to network formation at pH_G_. Ca^2+^ and H^+^ charge screening decreases the charge repulsion between the oligosaccharide side chains, exposing hydrophobic domains in mucin. Neutralization of the PGM side-chain charges is seen as the zeta potential remains around zero for pH values at and below the pH_G_. Note that, as expected at low pH values, the impact of the Ca^2+^ ions is somewhat mitigated, as there is already a large concentration of H^+^ ions available in solution. This charge neutralization and exposure of the hydrophobic regions allows for increased supramolecular bonding, hydrophobic effects, and chain entanglement, resulting in increased mucin-mucin interactions, larger moduli, and higher surface tensions in the gel state.

#### Chemical bonding characterization using spectroscopy

**Figure 11:**
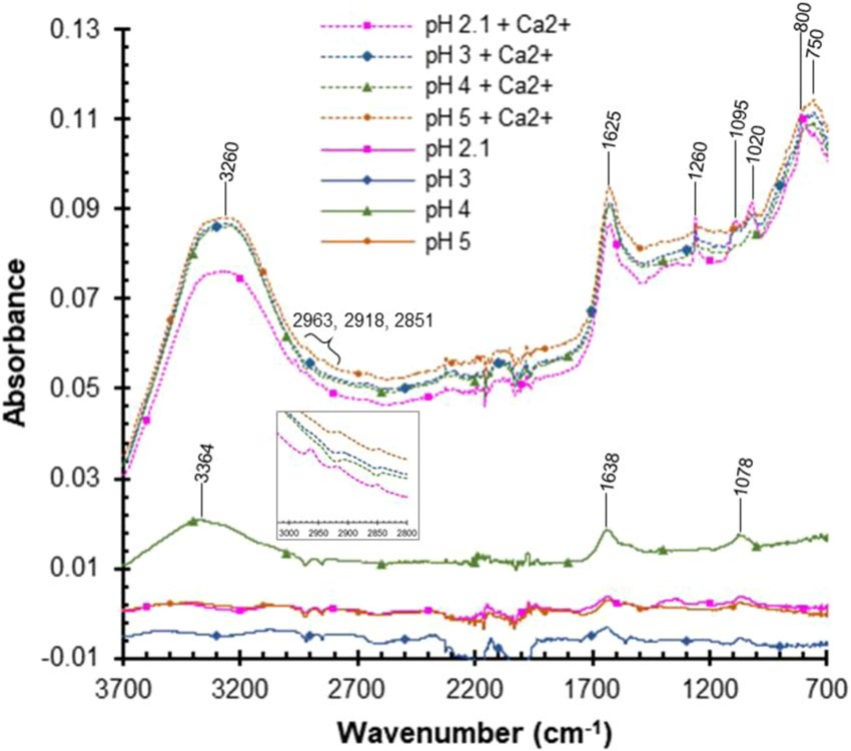
Representative FTIR spectra of 10 mg/mL PGM solutions at various pH values, both with (dashed lines) and without (solid lines) 10 mM CaCl_2_.

Gelation represents the formation of a supramolecular network between the mucin biopolymer chains in solution. As prior characterization showed that non-covalent crosslinking in PGM gel networks involves chemical interactions in addition to physical ones, FTIR spectroscopy was used to identify not simply the presence of functional groups, as mucin is a complex biopolymer, but the changes in functional groups exposed and bonding state with changes in H^+^ and Ca^2+^ concentration. FTIR spectra for the neat PGM solution samples, as well as mucin solutions with adjusted pH and CaCl_2_ addition, are presented in Fig. 11. Peaks were observed in the 3200–3300 cm^−1^ wavenumber range which are indicative of O-H stretching of the carboxylic acid. As pH is decreased (H^+^ is increased) and/or Ca^2+^ is added, the carboxylic anions (COO^-^) revert to carboxylic acid groups (COOH) causing the salt bridges in the aspartic acid and glutamic acid amino acids to fail, resulting in decreased electrostatic repulsion between the carbohydrate sidechains^13,63,64^. With these salt bridges no longer in place, the mucin strand unfolds exposing hydrophobic sites previously confined to the mucin strand interior. The exposure of these hydrophobic regions leads to hydrophobic effects involving the cysteine regions with an increase in mucin-mucin strand interactions and resultant longer-range structure and network formation. Peaks corresponding to C-H stretching were observed in the ~2950–2800 cm^−1^ range^65^. All samples also showed amide I (C = O) and II (C-N and N-H) peaks between 1640 and 1620 cm^−1^ although the peak maximum was shifted slightly to lower wavenumbers in the mucin samples with CaCl_2_ added^66–69^. For the mucin samples with CaCl_2_ added, strong peaks at 800 cm^−1^ and below were observed which may correspond to N-H out of plane bending or C-Cl stretching. Strong C-O stretching peaks at 1260, 1095, and 1020 cm^−1^ arising from the carbohydrate (oligosaccharide) groups were seen in samples with added CaCl_2_, with the most pronounced found in the pH 2 mucin solution with added CaCl_2_. This indicates an increase in C-O bonding between exposed glycosylated domains of mucin proteins, where “glycosylated domains” refers to regions with carbohydrate side chains that are covalently-bound to serine and threonine in the protein backbone^35^. Increased interactions between these glycosylated domains strengthen the mucin network through non-covalent crosslinking and long-range polymer-polymer interactions^35,70^. Disulfide bonding, also known to play a role in mucin network formation through end-to-end linkages, is expected in the wavenumber range of ca. 525 to 486 cm^−1^;^71–73^ however, absorbance could not be collected at wavenumbers below ~700 cm^−1^ with the ATR set-up used in this study^71,74^. The FTIR data show increased absorbances for both C-O bonding and glycosylation, providing evidence for this increased intramolecular bonding as a result of Ca^2+^ interactions with PGM.

## Materials and Methods

### Materials

Solutions of PGM mucin (Type II, Sigma Aldrich, CAS No. 84082–64–4) at 10 mg/mL were prepared using filtered ultrapure (ASTM Type I, 18.2 MΩ-cm, <5 ppb TOC) water. The PGM was used as received (unpurified). At 10 mg/mL, mucin solutions display diffusive behavior, allowing observation of pH and ion effects on gelling behavior to be decoupled from the effects of higher concentration^30,31^. For the rheology study, nitric acid and potassium hydroxide were used to form a series of PGM solutions at various pH values. Nitric acid is an oxidant and has been used to oxidize thiols to disulfide bonds^75^. The presence of nitric acid in low pH PGM solutions may have promoted additional cross-linking and stronger gel-like behavior than if a non-oxidizing acid were used to adjust pH^76^. Samples of PGM in water (neat) with an ‘as prepared’ pH value of 3.83 ± 0.03 were adjusted to three discrete pH values of 2.1, 4.0, and 5.8. In addition to these pH-adjusted PGM samples, a set of PGM solutions was prepared by adding 10 mM calcium chloride (via calcium chloride dihydrate, VWR International, ≥99%) to the aqueous PGM solutions at each pH value tested. PGM solutions were prepared at pH values of 2.1, 3.0, 4.0 and 5.0 in the same manner as described previously^77^. Note that in adjusting pH, the ionic strength of the PGM solutions was not held constant. To examine the impact of pH and calcium chloride concentration, the PGM solutions were characterized using zeta potential (ZP), dynamic light scattering (DLS) micro-domain particle sizing, and FTIR spectroscopy. All solutions were well-stirred over a 4–8 hour period, refrigerated overnight, and then allowed to warm to room temperature prior to characterization. Solutions were also refrigerated between sampling events. To obtain zeta potential and particle sizing data, the 10 mg/mL PGM solutions were stirred for 4–8 hours until homogeneous and were then diluted 100x to 0.1 mg/mL for the light scattering experiments.

### Rheology

Rheological measurements were collected with a TA Instruments Discovery Hybrid Rheometer II (DHR-2) using TRIOS software (v4.3). A 40 mm cone and plate geometry with 2.013° angle and a 53 µm gap was used for frequency and flow sweeps. The cone and plate geometry was chosen to maintain a constant shear rate throughout each sampling period. Rheological data were obtained using a solvent trap to discourage evaporation during each run and maintain proper filling of the sample liquid.

Frequency and flow sweeps were performed on six samples at three different pH values both with and without the addition of CaCl_2_. The effect of PGM concentration and temperature were also surveyed. Higher mucin concentrations (20–60 mg/mL) were shown to increase viscoelastic properties. However, increasing the temperature to a more physiologically relevant value of 37 °C resulted in a negligible change in rheological behavior.

To perform frequency sweeps, the linear viscoelastic region (LVR) was determined for each sample to ensure that the measurements are independent of imposed strain, as testing performed in this region yields more reliable and reproducible results^78^. The LVR is the range of shear stress values over which the storage modulus (G′) and the loss modulus (G′′) and other viscoelastic properties are independent of applied forces^79^. Frequency sweeps were performed in the LVR to ensure the mucin properties are independent of strain, allowing the viscoelastic moduli to be assessed consistently between samples. For LVR determination, an oscillating amplitude sweep was performed for each sample at a frequency of 1 rad/s over a stress range of 0.01 to 100 Pa. The LVR was determined by the average strain value that produced constant storage and loss moduli values (in the range of ca. 0.01 strain). Frequency sweeps (0.01 to 100 rad/s) were performed at each sample’s LVR average strain value for each sample at 25 °C. All error values presented are 95% confidence intervals.

Flow sweeps were performed on a range of shear rates from 0.001 to 100 s^−1^ at 25 °C with a sample period of 30 s for each data point collected. All error values for data are displayed or written as 95% confidence intervals.

### Dynamic light scattering micro-domain particle sizing

The Nanobrook Omni PALS instrument equipped with Brookhaven Instruments Particle Solutions Software was also used to perform micro-domain particle sizing via dynamic light scattering (DLS). DLS measurements were collected for the PGM solutions at discrete pH values of 2.1, 3.0, 4.0, and 5.0. Samples were filtered with a 1.2 µm syringe filter to remove large scale contaminates (e.g., dust) before DLS measurements.

### Zeta potential

A Brookhaven Instruments Nanobrook Omni phase analysis light scattering (PALS) instrument was used to perform zeta potential experiments. To obtain zeta potential data as a function of pH, a BI-ZTU 4-pump autotitrator unit was utilized along with a BI-ZELF flow cell. The PALS program within the Brookhaven Instruments Particle solutions software was used to control experiments and collect and analyze data. The flow cell was rinsed with ultrapure Type I water and emptied before each experiment. The pH of the sample was modified by the autotitrator with nitric acid and potassium hydroxide, and then zeta potential measurements were collected at each discrete pH value in order to determine the pH value for the point of zero charge (pzc).

### Surface tension

A Dynamic Contact Angle Measuring Device and Tensiometer (DCAT) 25 from Dataphysics was used to measure the surface tension of PGM solutions at discrete pH values of 2.0, 3.9, and 6.0. All measurements were taken using a Du Noüy ring geometry and data were analyzed using DCATS software and Microsoft Excel.

### FTIR spectroscopy

FTIR spectroscopy experiments were performed using a nitrogen-purged Thermo Fisher Nicolet iS50 instrument using a deuterated triglycine sulfate (DTGS) detector, attenuated total reflectance (ATR) accessory with diamond-ZnSe crystal and a XT-KBr beamsplitter. PGM solutions were each deposited dropwise onto the ATR crystal and spectra collected (with a minimum of 256 scans) using ambient air as background. Absorbance spectra were collected and analyzed using Thermo Scientific OMNIC software. Water spectra were collected and subtracted from each sample spectra to remove absorbance bands from the sample solvent.

## Conclusions

While other studies have examined the effect of concentration on the viscoelastic properties of mucin solutions^30,40,52^, in the present effort we have examined the effect of H^+^ and Ca^2+^ ionic conditions on PGM physicochemical properties through pH changes and CaCl_2_ addition. Structure-property changes related to the sol-gel transition for PGM were examined with regard to ion effects using rheology, light scattering, zeta potential, and surface tension studies. Solutions of porcine gastric mucin (PGM) were shown to exhibit a sol-gel transition that could be controlled by H^+^ ion concentration (pH) and addition of the divalent Ca^2+^ ion. All PGM solutions exhibited shear-thinning behavior with viscosity decreasing with increasing shear rate in flow sweep experiments. PGM solutions without CaCl_2_ addition displayed a sol-gel transition at ca. 4.0 pH and gel-like behavior at and below the gel point pH (pH_G_). When CaCl_2_ is added at and above the pH_G_, PGM solutions have higher viscosities, as well as higher storage and loss moduli. Conversely, when CaCl_2_ is added below pH_G_ (pH 2.1), the storage and loss moduli no longer display a clear sol-gel transition. At low pH, it is likely that the high concentration of H^+^ ions present result in charge screening which suppresses the impact of Ca^2+^ ion addition. Dynamic light scattering and zeta potential measurements not only support the PGM gelling behavior observed with rheology at low pH values (<pH 4), but also show that the addition of CaCl_2_ to PGM solutions increases the isoelectric point due to the neutralization of negative mucin surface charges by the Ca^2+^ ions. The larger effective mucin microdomain size in more acidic conditions supports the formation of a gelled mucin network and is corroborated by an increase in surface tension ≤ pH 4. To examine the chemical bonding changes involved in network formation, FTIR spectroscopy was used to highlight the role of Ca^2+^ in promoting mucin gelation through increased intramolecular bonding. The relationship between the viscoelastic behavior of mucin solutions and the dynamic mucin network structure is critical for predicting and controlling mucus properties. In addition, understanding how molecular-level functional group interactions impact the macroscale rheological behavior of mucin provides insight into a wide range of systems including polyelectrolyte solutions, structured fluids, and the application of therapeutics in physiological conditions with changing ionic conditions.
